# RDMA data transfer and GPU acceleration methods for high-throughput online processing of serial crystallography images

**DOI:** 10.1107/S1600577520008140

**Published:** 2020-07-31

**Authors:** Raphael Ponsard, Nicolas Janvier, Jerome Kieffer, Dominique Houzet, Vincent Fristot

**Affiliations:** a ESRF – The European Synchrotron, 71 Avenue des Martyrs, 38000 Grenoble, France; b GIPSALAB, Grenoble Alpes University, 11 Rue des Mathématiques, 38400 Saint-Martin-d’Hères, France

**Keywords:** online data processing, RDMA, RoCEv2, GPU, SSX, online data analysis

## Abstract

The continual evolution of photon sources and high-performance detectors drives cutting-edge experiments that can produce very high throughput data streams and generate large data volumes that are challenging to manage and store. In these cases, efficient data transfer and processing architectures that allow online image correction, data reduction or compression become fundamental. This work investigates different technical options and methods for data placement from the detector head to the processing computing infrastructure, taking into account the particularities of modern modular high-performance detectors. In order to compare realistic figures, the future ESRF beamline dedicated to macromolecular X-ray crystallography, EBSL8, is taken as an example, which will use a PSI JUNGFRAU 4M detector generating up to 16 GB of data per second, operating continuously during several minutes. Although such an experiment seems possible at the target speed with the 100 Gb s^−1^ network cards that are currently available, the simulations generated highlight some potential bottlenecks when using a traditional software stack. An evaluation of solutions is presented that implements remote direct memory access (RDMA) over converged ethernet techniques. A synchronization mechanism is proposed between a RDMA network interface card (RNIC) and a graphics processing unit (GPU) accelerator in charge of the online data processing. The placement of the detector images onto the GPU is made to overlap with the computation carried out, potentially hiding the transfer latencies. As a proof of concept, a detector simulator and a backend GPU receiver with a rejection and compression algorithm suitable for a synchrotron serial crystallography (SSX) experiment are developed. It is concluded that the available transfer throughput from the RNIC to the GPU accelerator is at present the major bottleneck in online processing for SSX experiments.

## Abstract 2

Evaluation of remote direct memory access data transfer from an X-ray detector head into a remote-processing graphics processor unit in the scope of a synchrotron serial crystallography experiment performed at 1000 images s^−1^.

## 1. Introduction   

Many X-ray experiments at synchrotron radiation and free-electron laser facilities already produce data streams at throughput rates that are beyond the capacity of classic computer architectures. The imbalance between the potential of analysis systems and the level of data flux issued from detectors is further increased by various synchrotron upgrades or enhancements in data-collection methods such as fine slicing and continuous acquisition (Willmott, 2019 ▸). New generation X-ray detectors have also become available, featuring larger sensor areas, higher dynamic ranges, higher pixel densities and faster acquisition rates. This leads to a big data challenge and enforces the use of innovative software and hardware (Leonarski *et al.*, 2020 ▸).

### 1.1. High-throughput online-processing bottlenecks   

The standard workflow (acquisition, transfer, storage of all data prior to batch processing) using new detectors working at full capacity and high-throughput networks will put a tremendous strain on computing and storage infrastructures. Currently, the bottlenecks are inside the computer systems themselves, on the data path from network card to processing cores and then to storage units.

Remote direct memory access (RDMA) is a solution that bypasses some of the major hardware and software bottlenecks in high-performance computing (HPC) systems. It offloads data movement from the central processing unit (CPU) and ensures direct placing of data to their final destinations, without extra copies.

This technology has attracted the interest of the detector community, especially since the RDMA over converged ethernet (RoCEv2) became available (Ibta, 2014 ▸).

In this article, we have chosen to use the new ESRF serial crystallography EBSL8 experiment as an example case for the challenges and methods investigated. This experiment will be installed in the ID29 beamline and is due to be operational by the end of 2021 (Coquelle *et al.*, 2015 ▸).

### 1.2. Synchrotron serial crystallography challenges   

**Figure 1:**
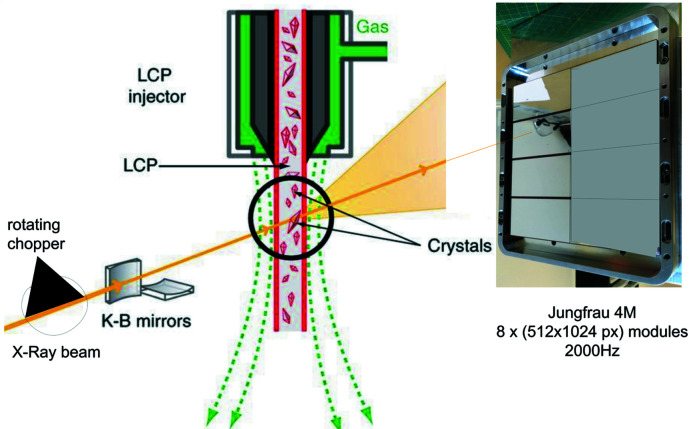
An example of a serial crystallography setup. The main components are the LCP crystal jet, the JUNGFRAU 4M detector and the rotating chopper (Source: Daniele De Sanctis, ESRF and Aldo Mozzanica, PSI).

Synchrotron serial crystallography (SSX) is among the most demanding cases of photon science in terms of data throughput. In a typical SSX experiment, a liquid crystalline polymer (LCP) jet propels microcrystal samples in a pulsed X-ray beam, as shown in Fig. 1 ▸. In the case of EBSL8, a rotating chopper produces X-ray pulses synchronous to the data-acquisition system. This enables the collection of alternate dark and signaling images at a maximum rate of 2000 images s^−1^. When acquiring 4M pixel 16-bit images, such a high repetition rate will result in a 128 Gb s^−1^ data stream. This will produce nearly 1 TB of raw data in 1 min. Continuous operation requires an efficient online data-reduction scheme.

### 1.3. The JUNGFRAU detector   

The JUNGFRAU detector (Mozzanica *et al.*, 2018 ▸) was developed at the Paul Scherrer Institut (PSI, Switzerland). It was initially designed for free-electron laser experiments but its characteristics are well adapted to other applications with pulsed beams, such as SSX experiments. A specific characteristic of the JUNGFRAU detector is automatic selection of the gain level for each individual pixel, depending on the signal detected. The photon count is derived from the energy deposited in each pixel, which has to be computed from the raw digital value by subtracting a pedestal offset of a previous dark image and dividing by a gain factor. Three different pedestal values and gain-factor values are needed for each pixel. Thus, this adds up to 24 million correction coefficients for a 4M detector. Hence, the processing of a single JUNGFRAU 4M raw-data frame to produce a final image requires 4 million 16-bit integer subtractions and 4 million 32-bit floating-point divisions.

### 1.4. Graphic processing unit accelerators   

The computationally intensive conversion of raw data produced by a detector like the JUNGFRAU detector and their subsequent online processing imposes the use of massively parallel computing engines. Graphic processing units (GPUs) are now routinely used in high-performance and scientific computing applications because of their superior performance and ease of use. These are the best candidates to treat the data streams produced in SSX experiments.

During the time slot available between two images, the GPU can perform the raw-data correction, followed by the image rejection or compression. However, to sustain this type of data treatment for a long duration, it is essential to transfer the detector data into the GPU memory continuously and to trigger the events required to synchronize computation and data flow. This is the purpose of the RDMA techniques investigated in this work.

### 1.5. Proof of concept   

Commercially available detectors commonly use either proprietary or 10 Gb ethernet links. As a proof of concept, we have emulated a detector with a Linux workstation using a 100 Gb s^−1^ ethernet RDMA network interface card (RNIC) from Mellanox Technologies. This test bench aims at exploring and leveraging RDMA/zero-copy techniques and online GPU data-processing capabilities.

Firstly, we verified that the RNIC was able to keep up with the expected throughput when transferring data to CPU memory. Then we proposed an efficient synchronization mechanism between the RNIC and the GPU. It appears that this transfer, from the RNIC to the GPU accelerator, is capped by the computer internal-bus throughput that might be lower than the 100 Gb s^−1^ of the network link.

During the time slot available between subsequent frames, we were able to perform the conversion of the JUNGFRAU detector raw data and the image rejection using a very basic Bragg’s peaks counting algorithm. As an alternative to image rejection, we implemented a compression algorithm into the compressed sparse row (CSR) sparse matrix format.

Finally, the processed data were stored in a CPU memory buffer. In the scope of this article, we do not address the challenge of high-bandwidth backup to non-volatile file systems.

## 2. High-throughput RDMA data transfer   

In a typical high-performance detector, readout electronics monitor the photon sensors and transfer acquisition data at high speed into an internal local memory. Such an embedded system uses field programmable gate arrays (FPGAs) with a limited power budget. These FPGAs are not well adapted to implement high-level network protocols, which require complex software stacks such as the transmission control protocol (TCP), featuring sophisticated error handling and automatic data re-transmission. Therefore, the detector electronics generally implement single-sided data transfer to the computing unit, such as the user datagram protocol (UDP).

### 2.1. Limitations of conventional data transfer   

The most recent generations of NICs support 200 Gb s^−1^ (Mellanox, 2019 ▸). However, when using the traditional software library (socket API), packet losses might be experienced and they are not dropped during the transmission phase but within the operating system. This is a well known flaw (Price, 2019 ▸), related to the inner complexity of the network stack. Many time-consuming tasks are executed under the hood: data copies from/to the user and kernel memory space, complex interrupt handling and context-switches, *etc*.

Fine-tuning of the operating system, *e.g.* increasing multiple buffer size, carefully allocating interrupt handling and disabling the Linux kernel from pre-emptively scheduling tasks onto the core dedicated to the receiving task, enables higher rates to be achieved (Marek, 2015 ▸), but the result does not scale well above 10 Gb s^−1^. Such a setup has been implemented in the field of X-ray science for the SLS detector software (Homs, 2019 ▸) and successfully tested with a PSI Eiger 500K detector featuring two 10 Gb s^−1^ ethernet links. It successfully aggregated 5 Gb s^−1^ on each link.

The data plane development kit (DPDK) (Intel, 2015 ▸) is an interesting framework which consistently implements these principles: the whole application code runs in user space and stays busy polling the NIC to achieve low latency.

### 2.2. Overview of DMA and RDMA   

In modern computers, DMA controllers are located in peripheral devices and handle data transfer to and from the main memory without CPU intervention, leaving it free for other tasks. Some application software configures the DMA controller with a list of source and destination memory descriptors. Their processing proceeds in two steps. First the translation of the virtual addresses given by the application into physical addresses used by the DMA engine, and then the memory buffer must be pinned. Indeed those physical memory pages can be scattered and one must prevent the memory management unit from moving them during a DMA transfer.

RDMA is the generalization of DMA between remotely connected computers equipped with dedicated NIC or FPGA boards. The distinct advantage of this data-transfer solution is to bypass the CPU and operating system and to move ingress data directly into their final destination. An overview of RDMA can be found in the work of Romanow & Bailey (2003 ▸), and performance and best-practice studies can be found in the work of MacArthur & Russell (2012 ▸). Many authors have investigated RDMA performance in the HPC field (Tsai & Zhang, 2017 ▸; Wang *et al.*, 2018 ▸) at research facilities. Mohr performed a comprehensive evaluation of the upgrade of the fast-acquisition system at CERN and showed the key advantages (Mohr, 2016 ▸).

Several RDMA implementations are available but only a few of them are compatible with the requirement for high performance over long-distance communication. The internet wide area RDMA protocol (iWARP) (Chelsio, 2019 ▸) is built on top of the TCP stack to ensure lossless transmissions on ethernet. We did not evaluate this solution as it relies on the TCP stack, which is incompatible with detector electronics. Interestingly, there are preliminary studies (Grant *et al.*, 2015 ▸; Lenkiewicz *et al.*, 2018 ▸) of a UDP/iWARP implementation which deserve further investigation.

#### 2.2.1. RDMA over converged ethernet   

Infiniband (IB) from Mellanox Technologies (now NVIDIA) was the first implementation of RDMA. It uses dedicated hardware: RNIC, switches and cables. RoCE is an alternate solution that makes IB compatible with already existing ethernet infrastructures.

There are two implementations of RoCE: RoCEv1 is non-routable by ethernet switches, while RoCEv2, which encapsulates the RDMA payload in a UDP/IP datagram, is routable at the price of a slight overhead.

The vendor roadmap favors a wider adoption of RoCE, promising RDMA performances and seamless integration into existing infrastructure (Eitan, 2018 ▸). *SoftRDMA* (Miao *et al.*, 2017 ▸) is a software implementation of RoCE on a standard NIC that may be valuable for low-cost solutions or research and development purposes.

#### 2.2.2. Feasibility of RoCE implementation in real detectors   

It is feasible to exploit RoCE features in the context of detector data transfer: an intellectual property core (IP) implementing a subset of the RoCEv2 core in FPGA was designed (Mansour *et al.*, 2018 ▸) and implements a parallel calculation of the RoCE invariant check redundancy code. To our knowledge, it was the first RoCEv2 IP publicly available until the recently announced Xilinx embedded RDMA-enabled NIC (ERNIC) (Xilinx, 2019 ▸).

A system on a chip or a dedicated integrated circuit may also be considered to implement the RoCEv2 protocol as in the work of Go *et al.* (2017 ▸) or such as the BlueField chip (Mellanox, 2018*a* ▸).

## 3. Evaluation of high-throughput data-transfer protocols   

This work is part of a wider RASHPA project, a data-acquisition framework optimized for 2D X-ray detectors that is suffciently generic and scalable to be used in a wide diversity of new high-performance detector developments (Mentec *et al.*, 2014 ▸).

With this target in mind, we conducted a benchmark campaign covering several protocols matching the following requirements: (i) one-way communication without acknowledgement of transfer completion, (ii) compatible with the existing ESRF network infrastructure (ethernet links and switches), (iii) suitable for readout-electronic FPGAs and (iv) featuring capability to control the data destination addresses in the receiver memory from the detector.

On the receiver side, we have carried out the necessary tuning of the operating system to guarantee proper operation at full throughput: checking IRQ (interrupt request) and CPU affinity (Mellanox, 2018*b* ▸), setting real-time scheduler attributes of the control process, and increasing the network buffer size.

### 3.1. Methodology   

Benchmarks of conventional non-RDMA and RDMA-based data-transfer software applications were carried out by transferring packets tagged with an incrementing packet-sequence number to detect packet drops at the destination. To estimate the highest achievable bandwidth, we decreased the throughput until we obtained a transfer without losses for a given transfer size. The three technology families evaluated were:

(i) Socket solution. This is the conventional method relying on the socket library. It has been widely used since the start of the internet and has continuously evolved and adapted over time but no longer suits new hardware. However, some optimizations are still possible by tweaking the operating system.

(ii) Hybrid solution. This improves conventional applications leveraging the messaging accelerator library (*Mellanox/libvma*, Mellanox Technologies), a software library which intercepts system calls to the socket API and transparently replaces them using RDMA operations.

(iii) Full RDMA solution, capable of reaching the target rate. We have evaluated three versions: *WRITE*, *SEND* and *RAW*. All are based on the *libibverbs* eponym API. Both *WRITE* and *SEND* packets conform to the RoCEv2 format but differ in the way they manage the destination memory address. In a *WRITE* operation, the destination address is chosen at the time of sending and embedded in the packet with the data. The receiver RNIC is then fully autonomous. On the other hand, *SEND* lets the data receiver resolve the memory address it writes data to, upon reception of said data. Some CPU effort is required; with the *RAW* API the RNIC hardware shall offload any incoming ethernet packet, according to configurable ‘steering rules’. This mode underexploits some of the RNIC high-level capabilities.

### 3.2. Benchmark of transfer protocols   

Our results confirmed that the socket API and the hybrid methods suffer from packet drops. The measured throughputs are also far below the theoretical expectations for these methods. This is detrimental to our study and enforces the use of RDMA.

#### 3.2.1. A socket and hybrid solution comparison   

Table 1 ▸ presents a comparison of UDP using classic socket API without (column 1: UDP socket) or with Linux kernel optimization (column 2: UDP + kernel tuning), and the accelerated code with the VMA library (*Mellanox/libvma*) (column 3: UDP-VMA).

The results are more reliable when sending data always to the same destination address than when sending data to 100000 different memory addresses. This is because of the limited size of the RNIC cache memory.

#### 3.2.2. RDMA solutions comparison   

Table 2 ▸ presents a comparison of RDMA-based solutions (column 1, RoCE-v2 *SEND*; column 2, RoCE-v2 *WRITE*; and column 3, *RAW*); these methods have been presented in Section 3.1. For RDMA transfer, we did not notice any packet drop and we measured similar performance close to the theoretical maximum.

For compliance with the RASHPA project, the *WRITE* method was chosen and used for the rest of the project. In this operation mode, the detector device must compute on the fly two lists of memory addresses for the source, and for the destination as well.

#### 3.2.3. System load during RDMA transfer   

In our tests, even with a RDMA transfer, one CPU core was permanently running at 100% because the main application is continuously polling the RNIC transfer completion queue in order to be notified of the end of transfer. An interrupt handler can take care of this task, notably decreasing the CPU usage at the price of a slight increase in latency. However, we also observed packet drops at the highest throughput values when using such an interrupt handler.

## 4. RDMA-assisted online GPU image processing   

Processing in real-time large datasets, such as those produced in SSX experiments, is a major challenge. This computationally intensive task would put a lot of strain on the CPU without hardware accelerators such as GPUs.

GPU accelerators efficiently handle massively parallel operations but are not fully autonomous. A CPU application is involved in orchestrating data transfers from the host CPU to the GPU device and back. Our control application also pre-launches the GPU kernels (code running on GPU) so that their execution begins as soon as the data are available. The actual execution is delayed until the occurrence of a RDMA transfer completion event. This mechanism is presented in the next section.

For the sake of efficiency, we have implemented a data-processing loop with a sequence of three operations described below:

(i) Data transfer to GPU memory through the peripheral component interconnect express (PCIe). While the delivery order of UDP packets is not guaranteed, no reshuffle step is required thanks to the *WRITE* API which specifies the destination memory address in each packet.

(ii) Conversion of the detector raw data (JUNGFRAU in our example) into floating-point values; this involves image rejection or compression.

(iii) Transfer of the computational results from the GPU to a large CPU memory buffer when the image is not rejected.

These sequences are *CUDA* streams that can be executed in parallel. This transforms the previous loop into a three-stage pipeline in which transfers overlap with computations.

### 4.1. GPU accelerator and RDMA synchronization mechanism   

**Figure 2:**
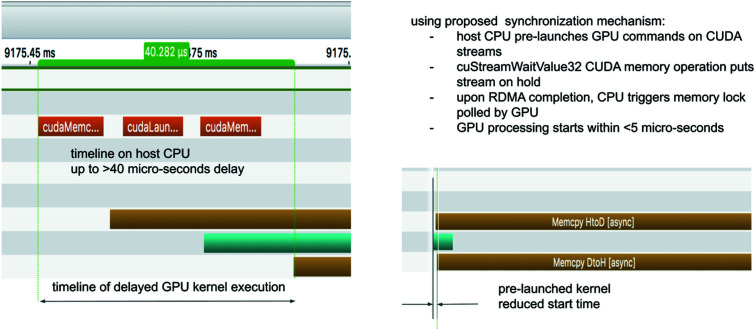
NVIDIA visual profiler snapshot of launch times. Implementing standard launches from the host upon completion (left) causes driver overhead in the range of tens of microseconds per operation. Using *CUDA* stream memory operation (right) decreases latency to a few microseconds.

Three heterogeneous systems are involved in our design: a RNIC, a CPU and a GPU. However, to date, a RNIC cannot directly trigger a GPU event so we have designed our own cascaded synchronization procedure described below:

(i) By design, the receiver CPU is not informed of the on-going RDMA traffic. However, it is possible to notify the CPU of an event: when a detector has sent a full image it uses a variant of the *WRITE* API that embeds a 32-bit event value in its payload.

(ii) Upon notification, the CPU application in turn triggers the GPU stream execution. A memory lock mechanism relying on *CUDA* stream memory operation (cuStreamWaitValue32) initiates, with a minimal latency, the actions previously put on hold in their streams. This mechanism removes the launch overhead detrimental to real-time data processing, as shown in Fig. 2 ▸.

**Figure 3:**
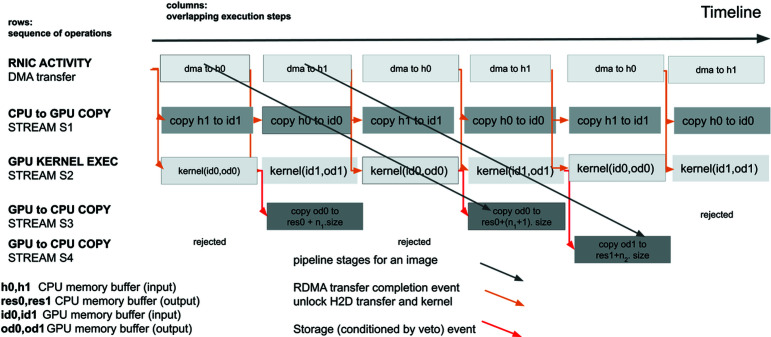
The GPU processing pipeline from top to bottom: DMA transfer, CPU to GPU transfer, data processing, GPU to CPU transfer. The pipeline involves three heterogeneous systems: RNIC, CPU and GPU. The RNIC cannot directly synchronize the GPU. Each stage in the execution pipeline is synchronized by an RDMA event or conditionally by the veto kernel result.

As shown in Fig. 3 ▸, the GPU application implements several double buffers so that the ongoing transfer is carried out in one buffer while computation is alternatively carried out in the second buffer. At the end of an RDMA transfer, a completion event triggers three simultaneous overlapping actions:

(i) The start of a CPU to GPU transfer data chunk #*n* + 2.

(ii) Consecutive kernel computations of data chunk #*n* + 1.

(iii) Transfer from the GPU to a large memory buffer on the CPU of the result of data chunk #*n* (if the image is not rejected).

When a non-pertinent image is rejected, the third step does not happen. Therefore, the result of the veto kernel algorithm shall conditionally trigger the transfer. As the condition is not evaluated prior to the launch, we use another stream memory operation lock triggered by the veto function. A credit-based algorithm controls the number of queued transfers, since the queue size is limited.

### 4.2. Development and evaluation test bench   

**Figure 4:**
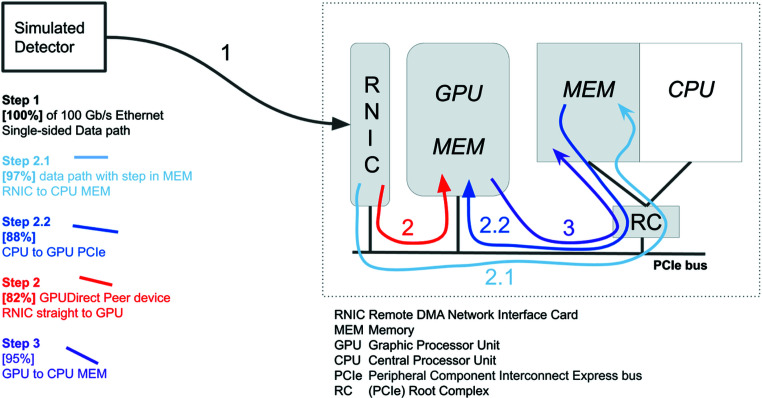
An overview of the maximum throughput in data paths (1, 2.1, 2.2, 3) inside a backend processing computer. Because of the lack of existing RDMA-compatible detectors, such a detector was simulated by a workstation. The processing computer embeds a NVIDIA QUADRO P6000 GPU accelerator. The GPUDirect technology (step 2) allows bypassing the extra copy in the central memory (steps 2.1 and 2.2).

The test bench compatible with high-throughput detectors has been upgraded with a GPU accelerator, as shown in Fig. 4 ▸.

### 4.3. NVIDIA GPUDirect evaluation   

**Figure 5:**
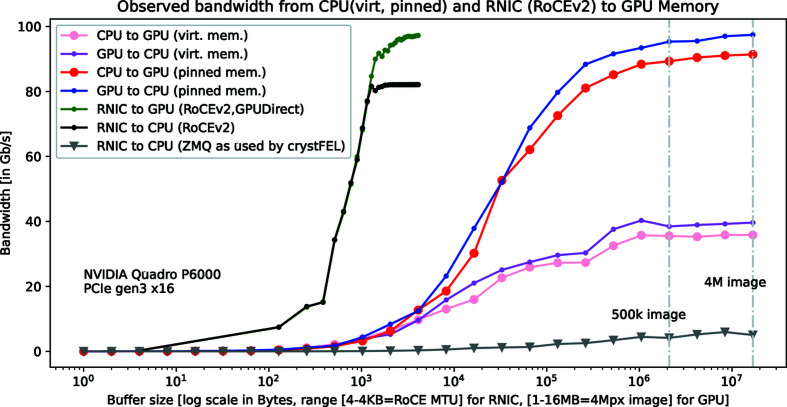
Transfer throughput from CPU and RNIC to GPU memory. The transfers from pinned memory are more efficient than from virtual memory. Using RDMA technology, full bandwidth was achieved from 1 KB. *ZeroMQ* transfers, which are used by the *CrystFEL* online reduction tool, are capped under 10 Gb s^−1^.

One key point for efficient GPU application is the optimization of the data-transfer bandwidth from CPU to GPU memory. Transfer speed from CPU virtual memory to GPU device memory is severely limited because movable memory must be pinned before DMA transfer, which takes time. However, allocating explicitly pinned host memory boosts speed, as shown in Fig. 5 ▸.

The GPU device memory may also be directly accessed by the RNIC (peer-to-peer DMA), removing the transfer step from CPU to GPU memory and decreasing latency. Quadro and Tesla NVIDIA GPUs feature this so-called GPUDirect technology, which was developed conjointly by NVIDIA and Mellanox Technologies (Shainer *et al.*, 2011 ▸).

It appears that GPUDirect throughput might be limited on some hardware by PCIe root complex poorly handling peer-to-peer transfer (Rossetti, 2014 ▸). This explains why NVIDIA is purposely integrating PCIe-switch chips into their high-performance DGX computers as a workaround for this issue.

The maximum possible speed for data processing is defined by the minimum of:

(i) RNIC to CPU speed, which is related to the length of the packet on the network (the width of the region of interest in the images that are sent).

(ii) CPU to GPU speed, which depends on the size and number of images processed in the GPU; it is wise to transfer either a large image or a bunch of small images.

For comparison purposes, Fig. 5 ▸ also exposes the performance of the *ZeroMQ* protocol, even if it relies on TCP/IP and therefore might not be easily embedded in a detector FPGA. The latest version of the *CrystFEL* software suite for SSX experiments has recently been upgraded with a *ZeroMQ/MessagePack* interface (White, 2020 ▸). *ZeroMQ* is an optimized socket library, originally developed for high-frequency trading applications where microseconds matter, and then largely reused by the scientific community (ZeroMQ, 2019 ▸). It appears that *ZeroMQ* is not well adapted to RDMA hardware and performances stay below 6 Gb s^−1^ depending on the selected messaging pattern: request/reply as in *CrystFEL*. Results are slightly better for push/pull patterns but are not much improved by the VMA library.

### 4.4. Detector raw-data conversion to float   

As stated in Section 1.3, the JUNGFRAU detector has three gain levels for each pixel, stored in two of the most significant bits of the raw data. The pixel value is stored in the 14 remaining bits as an integer. Therefore, the conversion to the integrated charge is 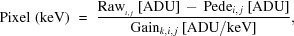
where *k* is the gain level, and the raw data and pedestal are expressed in arbitrary detector units (ADU).

This computation is inherently parallel. The gain factors are stored in a large constant dataset, *i.e.* they remain in the GPU memory, and only require one transfer from the CPU memory at the beginning of the process. Pedestal data are acquired interleaved with image data when the chopper is cutting the beam. These dark images are used to update the pedestal value for the highest gain. For the two other possible gain regimes, their pedestal value is expected to be constant.

### 4.5. Data rejection or compression   

In this work, our aim is not to propose any original or highly optimized algorithms but rather to assess a low-latency GPU/RNIC integration. Therefore, all the evaluated GPU kernels are extremely simple to allow a fast evaluation compatible with the 1 ms time slot counting the raw-data processing. More crystallographic sensible algorithms still have to be developed.

One simplistic rejection algorithm counts the intense pixels (considered as Bragg’s peaks) in each image, without noise correction or outlier removal. A first threshold defines the signal needed for a pixel to be counted as a Bragg’s peak, while a second threshold defines the minimum number of such pixels in an image to be accepted. Such counting has been implemented using atomic addition and shared memory.

As an alternative to rejection, we have implemented compression to the CSR matrix format using a classical parallel scan/reduction algorithm (Blelloch, 1989 ▸). The meta-programming capabilities of *PyCUDA* (Klöckner *et al.*, 2013 ▸) were used to generate the *CUDA* code of the cumulative-sum algorithm used in CSR matrix compression. The storage size of the result matrix was chosen *a priori* given the expected dataset sparsity.

It was not possible to use the dense-to-sparse conversion function provided in the NVIDIA *cuSPARSE* library. This highly efficient library has hidden memory allocation/deallocation, as emphasized in the work of Yang *et al.* (2018 ▸), preventing kernel execution from overlapping with data transfer.

Table 3 ▸ summarizes the kernel execution time observed with the NVIDIA visual profiler tool for the aforementioned algorithms.

## 5. Conclusions   

We have evaluated the feasibility of RDMA data transfer in the frame of high-performance X-ray detector development using gigabit ethernet data links after confirming the flaws of the standard socket API at 100 Gb s^−1^. It appears that one-way communication, a major challenge to stay compatible with detector embedded electronics, is possible using RoCEv2 unreliable datagram queue pair and *WRITE* verb API and commercially available RNICs in data-receiver computers.

The expected throughput is effectively achieved without usage of the destination CPU without packet drop and is solely limited by the RNIC packet per second message rates for small-size payloads.

The data flow could be dispatched to multiple data-processing accelerators in the destination computer because the memory address at destination is set by the detector source.

One can address online data-processing challenges, as encountered in SSX experiments, with GPU accelerators, for algorithms compatible with the reduced time slot available using a data-analysis pipeline and making simultaneous data transfers and computations.

We proposed a synchronization mechanism with the GPU accelerator that minimizes the kernel start latency using stream memory operations. Required events such as the end of RDMA transfer have been implemented using WRITE_WITH_IMM to notify the CPU application scheduling the processing pipeline.

Data transfer straight into GPU memory was evaluated with a GPU device featuring PCIe peer-to-peer capability (GPUDirect). Latency was decreased but throughput was also affected by hardware flaws.

Our work has also highlighted that the main limitation to performing online data analysis remains the data-transfer rate from PCIe to GPU memory (capped at 88 Gb s^−1^ using our hardware), as it is now slower than the network throughput. Release of new PCIes (generation 4) and other emerging high-speed GPU buses, such as NVLINK2, will contribute to a better exploitation of the full GPU processing speed.

Based on the outcome of our work, one can draw some conclusions, as far as the serial crystallography example featuring a JUNGFRAU detector is concerned: in the UDP-based design, 9 Gb s^−1^ data transfer is the limit, with unavoidable packet drops in continuous operation. In a first step of improvement, with a RDMA-compatible NIC, using the VMA library and without changing the application software, one can reach almost 26 Gb s^−1^. At the price of the rewrite of the application code with RDMA API, it is definitively possible to saturate a 100 Gb s^−1^ link without a packet drop.

The use of RDMA appears to be a very good solution to optimize the data transfer from the detector to a data receiver, especially the RoCEv2 technology preserving existing ethernet infrastructure. To our knowledge, only a few other actors (de Almeida *et al.*, 2018 ▸) are working on a RoCE implementation compatible with the requirements of X-ray detector devices.

### 5.1. Future work   

RoCEv2 routable properties make it also possible to fan out detector data to multiple-processing units for parallel processing, or the reverse – gathering in a single GPU accelerator the data flows from multiple detector modules. This functionality is a core feature of the RASHPA framework currently under development at the ESRF.

For more efficient low-latency GPU processing, we are considering minimizing the number of kernel launches with the use of continuously spinning GPU codes, as implemented for an adaptive optics controller in the work of Perret *et al.* (2016 ▸). We also plan to evaluate the new *CUDA* task graphs model for define-once-run-repeatedly execution flow (Ramarao, 2018 ▸) to decrease overheads associated with the submission of each GPU operation.

### 5.2. Methods and reproducibility   

Our benchmarks were run on a Dell T730 computer with two Intel Xeon CPUs E5-2643 v3 @ 3.40 GHz and 128 GB memory used as a detector simulator. The 100 Gb s^−1^ network card is a Mellanox ConnectX-5 EN in a PCIe generation 3 × 16 interface, which is directly connected by an optical fiber to the analysis computer. This network card behaves similarly to a standard NIC for TCP-UDP/IP but decodes RoCE datagrams and transparently offloads the Linux kernel (4.16.0-0.bpo.2-amd64) and the CPU. The Mellanox Technologies stack in use is OFED-4.4-1.0.0.

The data-receiver computer is a Dell T740 (4.16.0-0.bpo.2-amd64) with two Intel(R) Xeon(R) Gold 6134 CPUs @ 3.20 GHz and 192 GB memory, and a ConnectX-5 EN and OFED-4.6-1.0.1. The GPU is a NVIDIA Quadro P6000 with 24 GB memory in a PCIe generation 3 × 16 slot located on the same root complex as the RNIC.

Test images were extracted from a lysozyme dataset provided by Dectris (2019 ▸) to advertise the EIGER 4M detector for protein crystallography. Invalid pixels were removed from the images. All frames were processed to add a random pedestal and to apply a gain factor, and the gain bits have been set accordingly.

Three sets of data were generated for different detector sizes: 512 × 1024 pixels, 2048 × 2048 pixels and 128 × 8246 pixels (JUNGFRAU datagram format, four lines of 1024 pixels). All the datasets contain images and pedestals.

Application and GPU code are available from the ESRF GitLab repository, including Python scripts to produce datasets and measurements. A small subset of the code relies on an ESRF internally developed library *LIBDANCE* for communication and control of embedded systems but access requests from the scientific community are welcome.

## Figures and Tables

**Table 1:** Lowest observed bandwidths (in Gb s^−1^) causing packet drops with the usual (non-RDMA) protocol and the hybrid solutions when transmitting 10^7^ packets using Mellanox Technologies ConnectX-5 EN RNIC, either to one or many destination addresses (addr_num = 1 or 100000) Packets are not lost in the link during transfer but during Linux kernel UDP/IP stack processing.

	UDP socket		UDP + kernel tuning†		UDP + VMA	
addr_num:	1	1	1	100000	1	100000
Packet size (bytes)						
2048	7.2	6.0	10.9	11	26.7	26.1
4096	9.1	6.4	22.8	23.1	51.2	45
8246‡	9.8	5.2	36.7	27.4	77.0	39.7

† Increased system buffer.

‡ JUNGFRAU packet size.

**Table 2:** Observed bandwidths (in Gb s^−1^) from RNIC to CPU memory using RDMA The RoCE implementations use the RNIC capabilities efficiently. The *SEND* API is similar to a stream transfer. The *WRITE* API specifies, in addition, the destination memory address. The *RAW* API does not require a specific packet format.

Packet size (bytes)	RoCE-v2 *SEND*	RoCE-v2 *WRITE*	RDMA *RAW*
2048	94.5	95.2	33.3
4096	97.6	97.5	60.5
8246	N/A†	N/A†	99.1

† RoCE maximum payload (MTU) is 4096.

**Table 3:** GPU processing times on NVIDIA QUADRO P6000 of the proposed online algorithms, as shown using a NVIDIA profiler They are compatible with the available 1 ms time slot of the foreseen SSX experiment. For comparison with real-world use cases, we provide numbers for the yet to be published peak_finder algorithm from the GPU *pyFAI* suite. This algorithm implements the peakfinder8 algorithm from CrystFEL on GPUs.

	Kernel execution time (in nanoseconds for one image of the given size)			
Data batch (image + pedestal)	JUNGFRAU raw-data pre-processing†	Pre-processing + ‘simplistic’ Bragg’s peaks count†	Pre-processing + CSR matrix compression‡	Peak_finder (*pyFAI*)§
No. of image × size				
10 × 500K pixels	28	31	147	N/A
10 × 4M pixels	219	240	469	2167
10 × 2070 × 2167 (JUNGFRAU)	212	240	492	N/A

† Performed on a stack of images.

‡ Performed sequentially.

§ OpenCL implementation, for reference.
